# Impact of the Prognostic Nutritional Index on Postoperative Outcomes in Patients Undergoing Heart Surgery

**DOI:** 10.7759/cureus.43745

**Published:** 2023-08-19

**Authors:** Alaa A Almohammadi, Maha A Alqarni, Marwah Y Alqaidy, Sarah A Ismail, Reem M Almabadi

**Affiliations:** 1 Nutrition and Dietetics, King Fahad Armed Forces Hospital, Jeddah, SAU

**Keywords:** hospital, heart surgery, cardiovascular diseases, prognostic nutritional index (pni), malnutrition

## Abstract

Background

Malnutrition is associated with adverse outcomes in patients undergoing cardiac surgery. The prognostic nutritional index (PNI) is a validated tool for assessing nutritional status in cardiovascular diseases. This study aims to evaluate the prognostic value of PNI in heart surgery patients, including mortality rate, length of hospital and ICU stays, and infection rate, while investigating correlations with demographic and clinical characteristics.

Methods

A retrospective cross-sectional study was conducted in King Fahad Armed Forces Hospital in Jeddah, Saudi Arabia. Data from electronic medical records of patients undergoing heart surgery between 2019 and 2021 were retrospectively reviewed. The study involved patients with valvular heart disease, including those requiring concomitant procedures. Statistical analysis was conducted using t-tests, logistic regression, and Kaplan-Meier survival curve analysis.

Results

This study included 264 individuals with a mean age of 56.48±12.11 years. The prevalence of low PNI was 50.80% and high PNI was 49.20%. No significant differences in PNI levels were found between individuals with various clinical conditions, except for target vessel revascularization. The mortality rate was slightly higher in the low PNI group, but not statistically significant. Significant differences in laboratory findings were observed between high and low PNI groups. Individuals with low PNI had longer hospital stays.

Conclusion

Lower PNI levels consistently correlate with longer hospital stays and higher morbidity and mortality rates, suggesting the potential importance of PNI and other nutritional markers in assessing risk and predicting outcomes in cardiac surgery patients.

## Introduction

Despite recent technological advancements and novel surgical techniques, the risk of death and morbidity in patients undergoing heart surgery is still relatively high [1]. Several factors contribute to the mortality and morbidity rates, which encompass preoperative anemia, older age, coronary artery diameter, socioeconomic status, and left ventricular dysfunction [2-7]. Assessing the nutritional status of patients undergoing surgery is also extremely important. The presence of preoperative malnutrition has been linked to increased morbidity and mortality rates, prolonged hospital stays, and diminished post-surgery quality of life [8-10]. It exerts detrimental effects on various bodily systems, including the cardiovascular, immune, endocrine, and gastrointestinal systems. Additionally, malnutrition hampers the healing process during the recovery phase [11].

Various screening tools, such as the Mini-Nutritional Assessment and the Malnutrition Universal Screening Tool, are utilized to evaluate malnutrition in patients undergoing surgery [12,13]. Nevertheless, the utilization of these techniques in our routine clinical practice poses challenges due to their complexity and subjective nature [14]. Consequently, nutritional assessment is seldom incorporated into preoperative screening on a regular basis, and standardized approaches for evaluating the nutritional status of patients undergoing heart surgery have yet to be developed. The prognostic nutritional index (PNI) is a straightforward prognostic tool originally developed by Buzby et al. [15] and later modified by Onodera et al. [16]. It has been validated specifically in the context of cardiovascular diseases [17].

The PNI is readily obtainable and offers greater reproducibility compared to previous nutritional assessment tools. This is attributed to its calculation using objective laboratory test data. The calculation of the PNI involves evaluating the total lymphocyte count and serum albumin concentration, and numerous researchers have observed its effectiveness in assessing the perioperative immunological nutritional status and surgical risk for patients undergoing gastrointestinal, hepatic, and lung procedures. However, the applicability of PNI as a prognostic tool in heart surgeries has not been extensively studied [18-20]. This study aims to assess the prognostic value of PNI in heart surgery patients, specifically in predicting hospital mortality rate, length of hospital and ICU stays, and infection rate. Additionally, it seeks to compare the demographic and clinical characteristics of the patients with the PNI findings to understand the correlations between these factors and malnutrition.

## Materials and methods

Study design and data collection

A retrospective cross-sectional study was conducted in King Fahad Armed Forces Hospital between 2019 and 2021 to evaluate the correlation between PNI findings in heart surgery patients and hospital mortality rate, length of hospital and ICU stay, and infection rate. Data on patients from the electronic database of medical records was retrospectively reviewed. Preoperative data, including demographic characteristics, comorbidities, and laboratory findings, was extracted. Intraoperative and postoperative data, including type of procedure, length of ICU and hospital stay, mortality, and infection rate, were also obtained. Approval for this research was received from the Research Ethics Committee of King Fahad Armed Forces Hospital, where the research was conducted (REC 531 / Registration 2022-50).

Study settings and patients

Patients above 18 years old undergoing heart surgery at King Fahad Armed Forces Hospital between 2019 and 2021 were included in this study. The requirement for informed consent was waived because of the retrospective nature of the study. Patients who presented for surgery primarily due to valvular heart disease were enrolled, including those who required concomitant coronary artery bypass graft surgery, aortic procedures, or other cardiac procedures. Emergency operations and critical preoperative status were also included. Patients aged less than 18 years; undergoing transcatheter valve replacement, combined congenital heart surgery, or implantation of a ventricular assist device; or lacking data requirements for calculating nutritional indices or clinical outcome were excluded.

Nutritional assessment and classification

The patient’s preoperative nutritional status was determined using the PNI and calculated using the following equation:

10 x serum albumin (g/Dl) + 0.005 x total lymphocyte count (/mm^3^)

To define the cut-offs and how they have been calculated, patients were classified according to the PNI cut-off values obtained from receiver operating characteristic (ROC) curve analysis [21].

Statistical analysis

All the analyses and calculations were performed using Statistical Package for Social Science (SPSS, version 26; IBM Corp., Armonk, NY, USA). The normality of the continuous variables was checked using the Kolmogorov-Smirnov test. The data are presented as the means ± standard deviations for continuous variables and as the proportions for categorical variables. The chi-square test/Fisher exact test of association was applied to check the significant association between categorical variables like PNI. An independent sample t-test was used to compare continuous variables like age. The ROC curve was plotted to find the PNI cut-off value. A line graph was also plotted. Binary logistic regression was used to identify the relationship between the PNI and those associated risk factors that were significant in an independent sample t-test and chi-square test. The odds ratio (OR) and confidence interval (95% CI) were reported. A P-value less than 0.05 was considered statistically significant. Kaplan-Meier survival curve analysis was done. 

## Results

In the study, a total of 264 eligible participants were initially assessed for inclusion. After careful screening and evaluation, 264 participants were ultimately included in the study. There were no participants excluded from the study, as all eligible participants met the inclusion criteria and willingly participated throughout the entire duration of the research. Mean age of the participants was 56.48±12.11 years and there was higher proportion of males (73.50%) than females (26.50%). Overall mean PNI was 55.51±35.59. The percentage of patients with diabetes, hypertension, dyslipidaemia, and renal disease were 61%, 57.6%, 29.20%, and 11.20% respectively. The surgical site infection rate was 27.70% and there was 1.50% mortality in the study (Table 1).

**Table 1:** Demographic characteristics and medical history of individuals PNI: Prognostic nutritional index; COPD: Chronic obstructive pulmonary disease; CPAP: Continuous positive airway pressure, BIPAP: Bi-level positive airway pressure. CHD: Coronary heart disease; IHD: Ischemic heart disease; CA: Cerebrovascular accident; AF: Atrial fibrillation; CABG: Coronary artery bypass graft

Variable	Scale	Total (n=264) n (%), Mean± SD
Age (Years)		56.48±12.11
BMI (kg/m2)	Underweight	4 (1.50)
	Normal weight	60 (22.70)
	Overweight	89 (33.70)
	Obese	111 (42.00)
Gender	Male	194 (73.50)
	Female	70 (26.50)
PNI	Low	134 (50.80)
	High	130 (49.20)
Length of hospital stay (Days)	2019	15.03±8.98
	2020	13.53±6.77
	2021	15.37±9.20
Diabetes		161 (61.00)
Hypertension		152 (57.6)
Dyslipidaemia		77 (29.20)
Renal disease		30 (11.40)
Hypothyroidism		15 (5.70)
COPD		6 (2.30)
CHD		2 (0.80)
IHD		125 (47.30)
CA		1 (0.40)
AF		33 (12.50)
Aortic valve replacement		41 (15.50)
Mitral valve regurgitation		58 (22.00)
Mitral valves prolapse		39 (14.80)
Target vessel revascularization		27 (10.20)
CABG		181 (68.60)
Smoking		88 (33.3)
CPAP		19 (7.20)
BIPAP		60 (22.70)
Infection		73 (27.70)
Mortality		4 (1.50)

**Figure 1:**
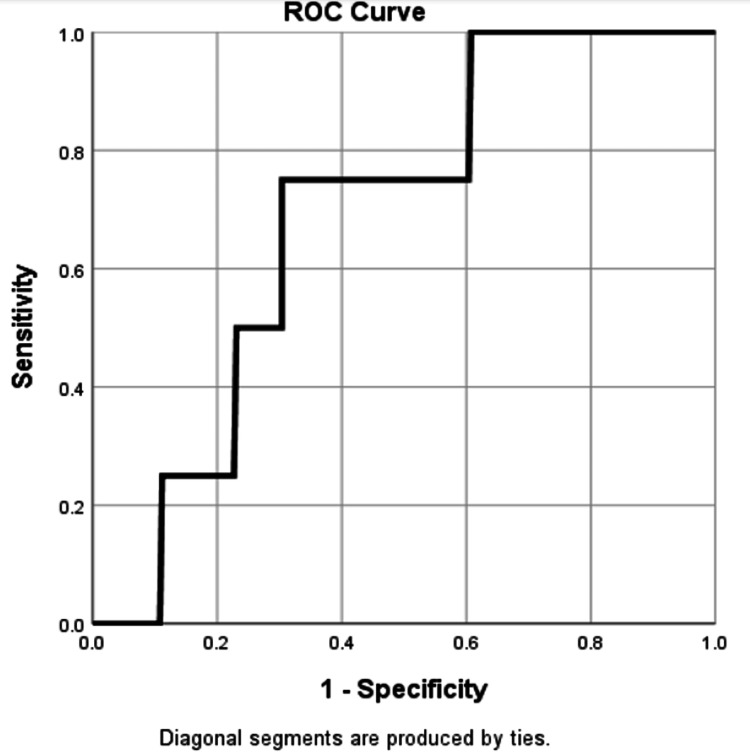
ROC Curve for PNI ROC: Receiver operating characteristics; PNI: Prognostic nutritional index

The ROC curve showed that PNI cut-off value was 53.27 following area under the ROC curve (AUC) 0.688, sensitivity 0.750 and specificity 0.319 (p=0.197). PNI greater than 53.27 was considered a high PNI and ≤ 53.27 was considered a low PNI (Figure 1).

There were significant differences found in PNI levels between individuals with and without target vascularization vessel (p = 0.024) and age (p = 0.032). The percentage of individuals who died during the study period was higher among those with a low PNI level compared to those with a high PNI level, although this difference did not reach statistical significance (Table 2).

**Table 2:** Comparison of baseline and demographic characteristics among PNI levels PNI: Prognostic nutritional index; COPD: Chronic obstructive pulmonary disease; CPAP: Continuous positive airway pressure; BIPAP: Bi-level positive airway pressure; CHD: Coronary heart disease; IHD: Ischemic heart disease; CA: Cerebrovascular accident; AF: Atrial fibrillation; CABG: Coronary artery bypass graft

		PNI level		p value
		Low	High	
Gender	Female	52 (29.21)	18 (20.93)	0.153
	Male	126 (70.79)	68 (79.07)	
Age (Years)	Mean ± SD	57.59 ± 11.52	54.19 ± 13.01	0.032
Diabetes	Yes	109 (61.24)	52 (60.47)	0.904
	No	69 (38.76)	34 (39.53)	
Hypertension	Yes	108 (60.67)	44 (51.16)	0.143
	No	70 (39.33)	42 (48.84)	
Dyslipidimia	Yes	47 (26.40)	30 (34.88)	0.155
	No	131 (73.60)	56 (65.12)	
Renal disease	Yes	21 (11.80)	9 (10.47)	0.749
	No	157 (88.20)	77 (89.53)	
Hypothyroidism	Yes	13 (7.30)	2 (2.33)	0.102
	No	165 (92.70)	84 (97.67)	
COPD	Yes	5 (2.81)	1 (1.16)	0.667
	No	173 (97.19)	85 (98.84)	
CHD	Yes	0 (0)	2 (2.33)	0.105
	No	178 (100)	84 (97.67)	
IHD	Yes	84 (47.19)	41 (47.67)	0.941
	No	94 (52.81)	45 (52.33)	
CVA	Yes	0 (0)	1 (1.16)	0.326
	No	178 (100)	85 (98.84)	
AF	Yes	24 (13.48)	9 (10.47)	0.487
	No	154 (86.52)	77 (89.53)	
Aortic valve replacement	Yes	28 (15.73)	13 (15.12)	0.897
	No	150 (84.27)	73 (84.88)	
Mitral valve regurgitation	Yes	37 (20.79)	21 (24.42)	0.504
	No	141 (79.21)	65 (75.58)	
Mitral valves prolapse	Yes	28 (15.73)	11 (12.79)	0.528
	No	150 (84.27)	75 (87.21)	
Target vessel revascularization	Yes	13 (7.30)	14 (16.28)	0.024
	No	165 (92.70)	72 (83.72)	
CABG	Yes	123 (69.10)	58 (67.44)	0.779
	No	55 (30.90)	28 (32.56)	
Smoking	Yes	53 (29.78)	35 (40.70)	0.078
	No	125 (70.22)	51 (59.30)	
Intubated	Yes	12 (6.74)	5 (5.81)	0.774
	No	166 (93.26)	81 (94.19)	
CPAP	Yes	11 (6.18)	8 (9.30)	0.358
	No	167 (93.82)	78 (90.70)	
BIPAP	Yes	40 (22.47)	20 (23.26)	0.887
	No	138 (77.53)	66 (76.74)	
Infection	Yes	49 (27.53)	24 (27.91)	0.949
	No	129 (72.47)	62 (72.09)	
Death	Yes	1 (0.56)	3 (3.49)	0.103
	No	177 (99.44)	83 (96.51)	

A comparison of high PNI and low PNI for laboratory findings showed that there was a significant difference in both groups with respect to haemoglobin, albumin, protein, lymphocyte, bilirubin, and alkaline transaminase (p<0.05) (Table 3). There was no significant difference found in mean PNI in three years (p = 0.402) (Table 4).

**Table 3:** Comparison of low and high PNI with respect to laboratory findings PNI: Prognostic nutritional index

Variables		PNI level		p- value
		Low Mean± SD	High Mean± SD	
Glutamic acid		9.61±10.85	8.26±3.74	0.273
Blood Urea Nitrogen		5.90±3.10	5.67±2.20	0.526
Creatinine		93.45±91.53	83.15±20.80	0.304
Haemoglobin		71.25±60.23	91.29±63.89	0.014
Sodium		136.28±3.25	136.81±3.57	0.233
Potassium		4.35±0.39	4.35±0.33	0.910
Albumin		37.80±3.50	49.90±55.98	0.004
Uric acid		373.01±114.79	366.08±94.30	0.655
C reactive protein-PRE		18.70±25.87	19.94±45.49	0.780
C reactive protein-POST		76.91±64.66	65.03±48.50	0.132
Protein		69.40±6.04	72.37±5.66	0.001
Cholesterol		3.98±1.19	3.70±1.17	0.084
Thyroglobulin		1.60±0.93	1.78±1.62	0.499
Lymphocyte		2.02±0.62	4.30±4.78	0.001
Haemoglobin A1c		7.52±5.71	7.54±1.94	0.978
Bilirubin		13.80±14.85	9.74±3.95	0.013
Alanine transaminase		27.11±20.28	35.34±25.16	0.005
Alkaline phosphatase		87.02±47.67	88.56±43.30	0.793

**Table 4:** Comparison of mean PNI in 2019, 2020 and 2021 PNI: Prognostic nutritional index

Year	PNI (Mean ± SD)	P-value
2019	57.60±43.26	0.402
2020	57.60±43.26	
2021	51.31±7.70	

The results of binary logistic regression showed a statistically significant relationship between high PNI and age, hemoglobin, protein, albumin, lymphocyte, bilirubin, and alkaline transaminase. A one-unit increase in age (OR: 0.977, 95% CI: 0.956-0.998, p = 0.032) and bilirubin (OR: 0.933, 95% CI: 0.886-0.983, p = 0.009) were associated with 0.977- and 0.933-fold increases in PNI respectively. Haemoglobin (OR: 1.005, 95% CI: 1.001-1.010, p = 0.015), protein (OR: 1.090, 95% CI: 1.040-1.142, p = 0.001), albumin (OR: 1.019, 95% CI: 1.257-1.541, p = 0.001), lymphocyte (OR: 9.303, 95% CI: 5.258-16.459, p = 0.001) and alkaline transaminase (OR: 1.016, 95% CI: 1.004-1.028, p = 0.008) were associated with 1.005-, 1.090-, 1.019-, 9.303-, and 1.016-fold increases in PNI, respectively. Only BMI was found to be an independent predictor of PNI. Individuals who underwent target vessel revascularization had an OR of 2.468 (95% CI: 1.104-5.515, p = 0.208) for having a high PNI level compared to those who did not (Table 5). 

**Table 5:** Predictor variable for the PNI based on the result of binary logistic regression PNI: Prognostic nutritional index; OR: Odds ratio; CI: Confidence interval; BMI: Body Mass Index.

Variable		OR	(95% CI)	P-value
Age (years)		0.977	(0.956-0.998)	0.032
BMI		1.004	(0.960-1.051)	0.851
Haemoglobin		1.005	(1.001-1.010)	0.015
Albumin		1.019	(1.257-1.541)	0.001
Protein		1.090	(1.040-1.142)	0.001
Lymphocyte		9.303	(5.258-16.459)	0.001
Bilirubin		0.933	(0.886- 0.983)	0.009
Alkaline transaminase		1.016	(1.004- 1.028)	0.008
Target vessel revascularization				
Yes		2.468	(1.104 – 5.515)	0.208
No		Reference		

**Figure 2:**
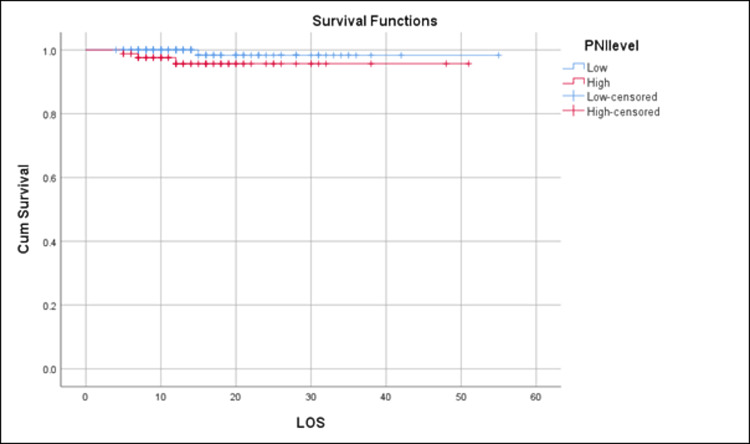
Kaplan–Meier survival curve analysis of total survival across the distribution of the prognostic nutritional index (PNI; Low and High) PNI: prognostic nutritional index, LOS: length of stay

Using the survival function, the distribution of hospitalization durations was analyzed and the proportion of patients still in the hospital at different time points was estimated. Through this, a comparison was drawn of the length of stay between patients with low and high PNI. Overall, the mean survival time for all individuals, regardless of PNI level, was 53.910 (SE = 0.569; 95% CI: 52.795 - 55.025) days. Based on these results, it appears that individuals with a low PNI level had a longer mean length of hospital stay than those with a high PNI level. Figure 2 shows that the mean survival time for individuals with a low PNI level was 54.333 (SE = 0.661; 95% CI: 53.038 - 55.629) days, while the mean survival time for individuals with a high PNI level was 49.168 (SE = 1.050; 95% CI: 47.111 - 51.266) days.

## Discussion

Various instruments have been developed for evaluating the nutritional status before surgery. Within the field of cardiac surgery, the Malnutrition Universal Screening Tool, Mini Nutritional Assessment, and Short Nutritional Assessment Questionnaire have emerged as standalone predictors that are linked to postoperative complications. Nevertheless, these assessment tools entail intricate scoring mechanisms and are susceptible to potential misinterpretations [12]. The notion of the PNI was first introduced by Buzby and colleagues in 1980. Later, Onodera and colleagues made modifications to the original PNI equation by incorporating serum albumin levels and peripheral lymphocyte count [16,21,22].

Different studies have categorized PNI values in diverse ways across the literature. In their original study assessing the role of nutritional assessment in gastrointestinal surgery patients, to allow statistical comparison, Buzby et al. classified patients as high risk (PNI ≥ 50%), intermediate risk (PNI = 40-49%) and low risk (PNI < 40%) [15]. Hayashi et al. evaluated the impact of the PNI on prognosis after cardiovascular surgery [23]. The subjects were categorized into two groups based on the PNI cutoff values, > 48 and < 48. Determination of the cut-off value was based on literature search and evaluating their own PNI data. The median value of PNI in their series was 48. Yoshihisa et al. studied the impact of nutritional indices on mortality in patients with heart failure. In their study, patients with a PNI >38 were considered normal, those with a PNI of 35-38 were considered to be at moderate risk of malnutrition and those with a PNI <35 were considered to be at severe risk [17].

Detsky et al. conducted a meta-analysis to evaluate the results of 18 controlled trials that measured the effectiveness of perioperative total parenteral nutrition (TPN) in improving major surgery outcomes [24]. They found that perioperative TPN reduced the risk for complications from major surgery (p = 0.21) and fatalities (p = 0.21). Poor nutritional status translates to lower PNI values. Available evidence suggests that decreased PNI can serve as a predictive indicator for postoperative complications and the overall prognosis following a surgical procedure [25]. In a previous study involving individuals with acute heart failure, it was established that PNI is independently associated with long-term survival. The researchers observed that the PNI reflected the presence of cardiac cachexia in heart failure patients, suggesting that the PNI could be considered an independent risk factor for mortality in individuals with heart failure [26]. Kwon et al. discovered that reduced PNI scores were associated with an elevated risk of one-year mortality and a composite outcome that included death, resuscitation or mechanical support, myocardial infarction, revascularization, new-onset atrial fibrillation, infection requiring antibacterial therapy, acute kidney injury, and stroke. Additionally, they observed an indirect impact of lower PNI scores on both outcomes independently [27]. In a recent investigation conducted by Tasbulak et al., it was found that nutritional indicators, including PNI, controlling nutritional status score (CONUT), and geriatric nutritional risk index (GNRI), were linked to mortality and long-term adverse cardiac and cerebrovascular events in patients undergoing isolated coronary artery bypass graft (CABG) procedures compared to the control group [22]. Published studies have also utilized the PNI to evaluate the risk in hemodialysis patients who are undergoing cardiac surgery [19]. Based on these findings, it is clear that application of these measures as predictors of prognosis in patients who have undergone CABG appears to be a feasible clinical practice option; however, there is still a lack of evidence supporting the usage of pre-operative PNI as a prognostic factor in cardiac surgery in general.

In our study, we aimed to investigate the PNI and its relationship with various factors, clinical conditions, laboratory findings, and patient outcomes. The findings suggested that PNI was influenced by age, hemoglobin, protein, albumin, lymphocyte count, bilirubin, alkaline transaminase, BMI, and target vessel revascularization. No significant differences were found in PNI levels between individuals with and without various clinical conditions. Laboratory findings demonstrated significant differences in haemoglobin, albumin, protein, lymphocyte count, bilirubin, and alkaline transaminase between the high and low PNI groups.

Studies have revealed that BMI, as well as albumin and prealbumin levels, have been identified as independent predictors of morbidity and mortality following CABG and valve surgery [28,29]. In this study, significant relationships between high PNI and age, hemoglobin, protein, albumin, lymphocyte count, bilirubin, and alkaline transaminase were revealed. BMI was identified as an independent predictor of PNI. These findings underscore the potential utility of these factors in predicting outcomes and assessing the risk in patients undergoing cardiac surgeries.

Our research suggests individuals with a low PNI level also had a longer mean length of hospital stay. Although the difference did not reach statistical significance, individuals with a low PNI level had a higher percentage of deaths during the study period compared to those with a high PNI level. Lower PNI levels have also been found to be strongly related to higher mortality and morbidity rates in recent cardiovascular disease studies [30-32]. Lee et al. [33] discovered that lower PNI may function as an independent predictor of early morbidity and mortality and that it was related to longer ICU and hospital stays. As per the findings of Hayashi et al. [23], they showed that surgical complications and survival were strongly correlated with a low prognostic nutrition index.

The present study possesses a number of potential limitations. Firstly, it's an observational and retrospective design, coupled with a small cohort size, which inherently restricts its scope. The limited cohort size prevented us from conducting meaningful subgroup analyses. To gain a comprehensive understanding, it is necessary to undertake larger-scale studies that include subgroup analysis of patients undergoing different types of cardiac surgery. Secondly, we were unable to establish the underlying pathophysiology of the relationship between the PNI and the other analyzed factors. Thirdly, our study was unable to confirm whether perioperative nutritional support improves clinical outcomes. To verify the practicality of the PNI and determine if preoperative nutritional support impacts clinical outcomes in patients with a low PNI, larger-scale randomized studies are needed.

## Conclusions

While our study revealed significant relationships between high PNI and various factors and laboratory findings, further research is needed to establish the broader applicability of pre-operative PNI as a prognostic factor in cardiac surgery. Nonetheless, lower PNI levels consistently correlate with longer hospital stays and higher morbidity and mortality rates in recent cardiovascular studies. These findings highlight the potential importance of PNI and other nutritional markers in predicting outcomes and assessing risk in cardiac surgery patients.
